# Desmoid Tumors Characteristics, Clinical Management, Active Surveillance, and Description of Our FAP Case Series

**DOI:** 10.3390/jcm9124012

**Published:** 2020-12-11

**Authors:** Lupe Sanchez-Mete, Virginia Ferraresi, Mauro Caterino, Aline Martayan, Irene Terrenato, Elena Mannisi, Vittoria Stigliano

**Affiliations:** 1 Gastroenterology and Digestive Endoscopy, IRCCS Regina Elena National Cancer Institute, 00144 Rome, Italy; elenamannisi@gmail.com (E.M.); vittoria.stigliano@ifo.gov.it (V.S.); 2 Department of Medical Oncology, IRCCS Regina Elena National Cancer Institute, 00144 Rome, Italy; virginia.ferraresi@ifo.gov.it; 3 Radiology Unit, IRCCS Regina Elena National Cancer Institute, 00144 Rome, Italy; mauro.caterino@ifo.gov.it; 4 Division of Clinical Pathology, IRCCS Regina Elena National Cancer Institute, 00144 Rome, Italy; aline_martayan@yahoo.it; 5 Biostatistics and Bioinformatic Unit-Scientific Direction, IRCCS Regina Elena National Cancer Institute, 00144 Rome, Italy; irene.terrenato@ifo.gov.it

**Keywords:** desmoid tumors, surveillance protocol, prognostic factors, CT scan, MRI, tumor growth, familial adenomatous polyposis (FAP)

## Abstract

(1) Background: desmoid tumors (DTs) are common in patients with familial adenomatous polyposis (FAP). An active surveillance approach has been recently proposed as a valuable alternative to immediate treatment in some patients. However, no clear indication exists on which patients are suitable for active surveillance, how to establish the cut-off for an active treatment, and which imaging technique or predictive factors should be used during the surveillance period. (2) Results: we retrospectively analyzed 13 FAP patients with DTs. A surveillance protocol consisting of scheduled follow-up evaluations depending on tumor location and tissue thickening, abdominal computed tomography (CT) scan/Magnetic resonance imaging (MRI) allowed prompt intervention in 3/11 aggressive intra-abdominal DTs, while sparing further interventions in the remaining cases, despite worrisome features detected in three patients. Moreover, we identified a possible predictive marker of tumor aggressiveness, i.e., the “average monthly growth rate” (AMGR), which could distinguish patients with very aggressive/life-threatening tumor behavior (AMGR > 0.5) who need immediate active treatment, from those with stable DTs (AMGR < 0.1) in whom follow-up assessments could be delayed. (3) Conclusion: surveillance protocols may be a useful approach for DTs. Further studies on larger series are needed to confirm the usefulness of periodic CT scan/MRI and the value of AMGR as a prognostic tool to guide treatment strategies.

## 1. Introduction

Desmoid tumors (DTs), also referred to as aggressive deep-seated fibromatosis, are slow-growing, non-metastasizing, benign, monoclonal fibroblastic tumors, which are rare in the general population, and common in patients with familial adenomatous polyposis (FAP; approximately 5–10% of all DT cases) [1,2]. The main differences between sporadic and FAP-related DTs are summarized in Table 1. Treatment options depend on location and clinical behavior, and include surgery, radiotherapy, or systemic treatment; in the last few decades, an active surveillance approach has been proposed as an alternative to more aggressive interventions [3,4]. However, the optimal therapeutic strategy for DTs is still under discussion; there is no standardization of surveillance protocols and no clear indication exists on prognostic or predictive biomarker for tumor progression [3,4].

In this paper, we provide an overview of DT characteristics and clinical management. We also report the characteristics of a series of FAP patients treated in our center, which prompted some evaluation on the feasibility of surveillance protocols for DTs and some preliminary assessment of possible markers for disease aggressiveness.

## 2. Epidemiology

In the general population, DTs are rare, accounting for 0.03% of all neoplasms and <3% of soft tissue tumors. They have an estimated incidence of 2–5 cases per 1 million of inhabitants in European countries, with a median age at diagnosis of 35 years [1,2,5,6,7,8].

It is estimated that patients with FAP have an approximate 800–1000-fold increased risk of developing DTs, compared with the general population [1,5]. DTs occur in approximately 7.5–15% of patients with FAP, although their prevalence could reach up to 21–35% given their pleomorphic presentation, which may lead to underestimated predictions [9].

## 3. Clinical Characteristics

Clinical features of DTs are characterized by location, size, number of lesions, and growth pattern. Morbidity and mortality mainly rely on location and growth pattern/clinical behavior.

In terms of location, DTs may affect soft tissues in all sites. They are classified as intra-abdominal DTs (IAD), when they develop in mesenteric folds or in retroperitoneal tissue in the small bowel, as abdominal wall DTs (AWD), when they form inside the abdominal wall or extra-abdominal DTs (EAD), when they originate from muscles located in the trunk, shoulder, neck, or extremities [1,5].

Growth patterns can be heterogeneous and vary from aggressive growth (10%), to a prolonged stabilization (50%) or spontaneous resolution (10%) [10,11]. This feature, along with location, size, and number of lesions, affects the onset (appearance) of symptoms and possible life-threatening complications [12]. In particular, IAD can cause a small bowel obstruction, ureter compression, and hydronephrosis, ischemic lesions, and severe complications, such as abscess formation, digestive hemorrhage, intestinal perforation, or fistulas [1,12].

Population-based studies on large case series [13,14,15] comparing sporadic and FAP-associated DTs revealed distinct clinical characteristics.

The authors observed the following main features: a predominance of DTs in females compared with males, especially in sporadic cases (63.1–73.2%) than in FAP-related ones (45.4–54%), a significant younger age at diagnosis in FAP cases (30.5–36 years) than in sporadic ones (41.6–42.3 years), except for the Mayo clinic series, in which age at onset was similar in both groups, and a higher prevalence of intra-abdominal location in FAP (51–68%) and extra-abdominal/abdominal wall sites in sporadic cases (50–89.1%). Less striking differences, represented more in FAP than in sporadic DTs, are a larger size (≥10 cm), have a multifocal pattern of distribution and a more aggressive clinical behavior [13,14,15,16]. Patients with FAP-associated DTs have a higher risk of recurrence (44% vs. 25%) and mortality (14% vs. 0%) compared with those affected by sporadic DTs [13]. Despite their histological benignity, DTs are the second/third leading cause of death in FAP patients who undergo prophylactic colectomy (overall mortality ranging from 10% to 50%) [17]. Notably, IAD location is associated with a significantly poorer survival. The propensity for intra-abdominal site in FAP could partially explain the mortality rate. Quintini et al. reported additional poor prognostic factors: tumor size > 10 cm (hazard ratio (HR): 1.44), presence of severe pain/narcotic dependency (HR: 2.22), and need for total parenteral nutrition (HR: 3.29) were found to negatively affect survival in the multivariate analysis (*p* < 0.001) [18].

## 4. Etiology and Predisposing Factors

The exact etiology of DT is unknown, but it is believed to be multifactorial with genetic, endocrine, and physical factors playing a crucial role in tumor development and growth [9,17,19,20,21,22].

Pregnancy has been reported to be a predisposing factor in 12–33% of cases, especially in sporadic cases (21% vs. 0% in FAP) [13]. Conversely, a previous surgical trauma is the main risk factor in FAP versus sporadic cases (64–83% vs. 13%, respectively) [17,20,22,23]. However, incidental desmoid tumors are also reported at index surgery in FAP DT [24].

To date, it is still unclear if the type of surgical procedure (total colectomy vs. proctocolectomy) or surgical approach (laparoscopic vs. open) can influence the development of DTs. Saito et al., in a retrospective study on 277 FAP patients, reported 14.1% of DTs, developed during a mean period of 26.3 months from surgery, and observed a significant difference in the risk of developing DTs in the 5 years after surgery depending on the type of surgery, but not on the approach (proctocolectomy was an independent risk factor, HR: 2.2; *p* = 0.03) [21]. Conversely, in a retrospective study in 672 prophylactic colectomies (602 open vs. 70 laparoscopy), Vitellaro et al. detected a lower risk of the development of DTs with the laparoscopic approach (estimated cumulative risk 4% vs. 13%; *p* = 0.042) [23].

Most DTs arise sporadically and are associated with somatic mutations in the β-catenin gene (*CTNNB1*) [25]. Some have been linked with pregnancy (high estrogen states) and previous trauma, and others are related to hereditary cancer syndromes, such as in the FAP [1,5] variant, the Gardner’s syndrome, in which polyposis is associated with prominent extracolonic features, including osteomas and multiple skin and soft tissue tumors (i.e., desmoid tumors), which can be the initial presenting findings, especially at extra-abdominal sites [26,27].

Both the *CTNNB1* mutation, which is mainly reported in somatic DTs, and mutations in the adenomatosis polyposis coli (*APC*) gene, which is the characteristic germline mutation of FAP syndrome, affect the Wnt signaling pathway. By promoting the expression of β-catenin and subsequent activation of the transcription factors of the T-cell factor/lymphoid enhancer factor (TCF/Lef) family, these mutations eventually modulate the expression of Wnt target genes, influencing proliferation, differentiation, migration, and apoptosis of cells [28]. Moreover, mutations in *APC* lead to alterations in intracellular adhesions and the destabilization of the cytoskeleton, which are responsible for the genesis of colorectal cancer, one of the main signs of FAP syndrome [29]. Some specific genetic mutations in *APC*, especially between codons 543–713, 721–972, and 1256–2011, have also been associated with an increased risk of FAP-associated DTs, highlighting the importance of appropriate surveillance protocols tailored to the patients’ characteristics [30]. In particular, in a meta-analysis on risk factors predicting desmoid occurrence in patients with FAP, Sinha et al. found that in a total of 4625 FAP patients—559 (12%) of whom developed DTs—a positive family history was the most significant risk factor (odds ratio (OR) 7.02, 95% CI: 4.15–11.9, *p* < 0.001), followed by *APC* gene mutation 3′ to codon 1399 (OR: 4.37, 95% CI: 2.14–8.91, *p* < 0.001) and previous abdominal surgery (OR: 3.35, 95% CI: 1.33–8.41, *p* = 0.01) [17].

## 5. Treatment Options

Choosing the optimal therapy for DTs is rather complex, as the disease is rare, and management varies depending on tumor size, location, symptoms, and disease growth pattern.

### 5.1. Surgical Resection

Before the year 2000, complete surgical resection with wide margins was considered the best course of treatment, especially in patients with AWD or EAD [5,31]. In the last few decades, multiple retrospective studies have highlighted the limitations of this approach, which include contraindications, risk of recurrence independently from the quality of surgical margins and risk of de novo DTs [32,33,34]. Different studies suggest that, despite wide excisions with negative margins being the goal of surgery, the risk of recurrence is independent from the achievement of microscopically clear resection margins or adjuvant therapy [33]; recurrence is modest even in the presence of positive margins (fewer than half of the patients, according to Mullen et al. [32]). Salas et al. also found that age, tumor size, and tumor site, but not surgical margins, were independent prognostic factors for patients with DTs; based on this observation, patients could be divided in separate subgroups, which might benefit from different therapeutic strategies [35]. For example, the risk of relapse seems to be correlated with the primary site of origin, with AWD a better prognosis than IAD and extremities desmoids. In addition, Bonvolot et al. reported a growth arrest in two-thirds of non-operated patients with EWD, suggesting that a nonaggressive, wait-and-see policy could be the best therapeutic strategy in this subgroup of patients [36].

A large, prospective study of the French Sarcoma Group was conducted on 771 patients with DTs to analyze the role of a wait-and-see approach versus immediate radical surgery. The relapse-free survival at 2 years was similar between the two groups (53% for surgery and 58% for observation only, *p* = 0.415); at univariate analyses, primary location proved to significantly influence the outcome. Favorable locations (abdominal wall, intra-abdominal, breast, digestive viscera, and lower limb) were associated to a 2-year event-free survival similar between surgery (70%) and the wait-and-see approach (63%; *p* = 0.413). In patients with unfavorable locations (thoracic wall, head and neck district, superior limbs), the 2-year event-free survival was significantly improved in patients initially managed with the wait-and-see approach (52%) compared with those who underwent initial surgery (25%; *p* = 0.001) [37].

The same considerations can also be applied for patients with FAP-associated DTs with similar progression-free survival at 10 years (33% and 49% for wait-and-see approach and surgery, respectively, *p* = 0.16) and with better outcomes for extra-abdominal and abdominal wall desmoids (63%) [21,38,39,40,41].

Moreover, surgery is usually contraindicated in IAD, due to the involvement of the small bowel, mesentery, and great vessels, the significant morbidity in case of extensive resection and the risk of possible complications, such as ischemia, fistula, hydronephrosis, and bowel obstruction [1,5]. In addition, DTs (and especially IADs) have a tendency to recur, even after complete surgical resection; mean time to recurrence is approximately 18 months (range: 4 months to 12 years) [42] and a second procedure is required in 75–85% of the cases [43]. Lastly, data show that surgery may be a risk factor for the development of de novo DTs, which were observed in 12% of the cases after restorative proctocolectomy, 13% after ileorectal anastomosis, and 13.5% after partial colectomy [44]. For these reasons, surgical treatment of DTs is currently highly debated, and a surveillance approach was proposed as more feasible in patients with FAP-associated DTs or in large slow-growing DTs that are involved in the mesentery, vessels, and organs [36,45,46].

### 5.2. Local Non-Surgical Treatment Options

Moderate-dose radiotherapy (RT) may be an effective option for patients with EADs only when surgical resection is not feasible for DTs in critical anatomical structure (head and neck district, shoulder girdle, etc.), and when potentially active systemic treatments are not available or contraindicated for co-morbidities and/or very advanced age (see systemic treatments) [19,47,48,49]. Meta-analyses and current guidelines do not confirm its role as post-operative treatment even in patients with R1 resection [31,50].

Among local treatments, isolated limb perfusion (with Tumor necrosis factor α (TNF-α) and melphalan) and cryoablation proved to be effective in selected cases treated in specialized referral centers [51].

### 5.3. Subsection

Upfront systemic treatment may be an option for patients with symptomatic progressing DTs, especially when surgery is associated to high morbidity and loss of function [31].

#### 5.3.1. Nonsteroidal Anti-Inflammatory Drugs

Nonsteroidal anti-inflammatory drugs (NSAIDs) have been suggested for the treatments of DTs based on the high expression of cyclooxygenase (COX)-1 and COX-2 observed in desmoid cells in vitro [52]. Literature data reported positive results with NSAIDs, such as sulindac or indomethacin, achieving stable disease or partial/complete responses in 37–57% of treated patients [53,54,55,56]. However, most of these results come from case reports and should be investigated in larger clinical trials.

#### 5.3.2. Hormone Therapy

Anti-estrogen therapy, mainly based on tamoxifen or toremifene, has proven to be effective in treating DTs, probably because of the strict association between tumor growth and endogenous hormonal environment [55,57]. The use of endocrine therapy (alone or in association with NSAIDs) should be reserved to oligosymptomatic patients with tumors located in non-critical anatomic sites. The low rate of response and a lack of a clear association with symptoms changes, together with the known side-effects of tamoxifen, including a higher risk of endometrial cancer, are factors to be taken into account and make anti-estrogen therapy not highly recommended. Interestingly, a study by Libertini et al. on 32 patients with aggressive fibromatosis treated with tamoxifen, reported no correlation between worsening or improvement of patient symptoms and disease response measured by Response Evaluation Criteria in Solid Tumors (RECIST)1.1 or MRI; thus, suggesting the need for prospective studies, focusing especially on patient symptoms [1,58].

#### 5.3.3. Chemotherapy

In the case/eventuality of symptomatic disease or failure of endocrine/NSAIDs approaches, clinical evidence indicates that chemotherapy can be effective in the management of DTs. “Low-dose”, weekly chemotherapy regimens, including methotrexate and Vinca alkaloids (vinblastine or vinorelbine), are considered the preferential approach in consideration of the relative low toxicity and respect of patient quality of life with a clinical benefit in approximately 80% of patients [59]. The combination of methotrexate and Vinca alkaloids also reported positive results in a group of 26 pediatric subjects, with tumor regression or stable disease achieved by the majority of the patients and no-to-moderate toxicity [60].

In case of highly symptomatic disease or critical primitive anatomic sites that require a fast-clinical response (e.g., FAP-associated DTs or head and neck DTs), anthracycline-based schemes can be taken into consideration [44,61,62,63]. In a study conducted in seven patients with symptomatic unresectable DTs who did not respond to previous hormonal therapy, the combination of doxorubicin and dacarbazine, followed by meloxicam proved safe and effective, with three complete responses, all patients achieving a significant clinically and radiologically tumor regression, and no major safety concerns [44]. Pegylated liposomal doxorubicin also appeared as a valuable single-agent approach, as shown by Constantinidou et al., in a report on 12 patients with aggressive fibromatosis. Pegylated liposomal doxorubicin reported an acceptable toxicity profile and a highly promising clinical activity, with 36% of the patients achieving objective response, seven cases of stable disease and important clinical benefits, such as pain relief, improved mobility, or cosmesis obtained by 11 out of 12 patients [61]. The superiority of anthracycline-containing regimens also emerged from the analysis of 62 patients with recurrent and/or unresectable DTs registered in the French Sarcoma Group, who presented variable tumor location and different previous systemic treatment failures. Indeed, anthracycline-based regimens reported a higher response rate compared with non-anthracycline regimen, such as methotrexate–vinblastine (54% vs. 12%) [62].

#### 5.3.4. Targeted Agents

Several tyrosine kinase inhibitors proved to be active in DTs.

Imatinib is a selective tyrosine kinase inhibitor that showed positive results in two phase II trials in patients with DTs who were not eligible for surgery. The first study showed objective response rates (ORR) in 6% of the patients (three out of 51) and 66% progression-free survival at 1 year [63], whereas the second one registered three cases of ORRs out of 41 patients, and 95% progression-free survival after 2 years [64].

Sorafenib, another tyrosine kinase inhibitor active in VEGFR, PDGFR, RAF, and RET, proved to be active in DTs [65]. In 2018, the positive results of the phase III trial, ALLIANCE, comparing sorafenib with placebo in patients with progressive, symptomatic, or relapsing DTs were published [66]. Median progression-free survival at 2 years was 81% versus 36% for patients receiving sorafenib or placebo, respectively, with an overall response rate of 33% and 20%, respectively.

Finally, pazopanib proved to be active in DTs in a phase II trial (PAZOPANIB Efficacy and Tolerance in Desmoids Tumors (DESMOPAZ) study) versus chemotherapy with methotrexate and vinblastine showing a 6-month non-progression rate of 80% vs. 50%, respectively [67].

## 6. Surveillance and Prophylactic Measures

In the last few decades, the routine use of aggressive first-line treatments for DTs, such as surgery and RT, has been progressively challenged by many authors [3,31]. Considering the unpredictable behavior of DTs and the high recurrence rates registered after surgery, close observation is now considered an acceptable approach, especially for asymptomatic patients; whereas treatment is recommended in symptomatic patients, in case of tumors adjacent to critical structures or for cosmetic concerns [8,19,68]. In patients with FAP, specific attention should be given to high-risk mutations (from codon 1444 to 1578) and a positive family history of DTs [69]. In addition, given that surgical trauma is a known risk factor for DTs in this population, prophylactic colectomy should be delayed if possible, or different approaches should be chosen over open surgery, if feasible [70].

Some authors have proposed treatment algorithms for newly diagnosed DTs in which the front-line consists of a conservative watchful waiting approach, and more aggressive treatments are postponed whenever possible [3,31]. The cut-off for an active treatment should be defined based on multiple variables, such as tumor size, growth rate, anatomical location, risk of organ damage or worsening of function [31]. In 2013, Crago et al. proposed a postoperative nomogram to estimate the risk of local recurrence after tumor resection. The nomogram was built on three main variables: tumor size, tumor location, and patient’s age, and could represent a useful tool to distinguish between patients who should be addressed to surgery after diagnosis and those in which alternative approaches (systemic treatment, radiations, observation) could represent a more feasible approach [71]. More recently, a study on patients with primary DTs in multiple anatomical locations identified pain (32%), progression (31%), or both (13%) as the main indicators for treatment requirement in patients undergoing a surveillance protocol. The study also confirmed that active surveillance is an appropriate strategy in patients with DTs, although almost half of the subjects may eventually require treatment [4].

Despite the increasing interest on surveillance protocols in DTs, a standardized approach is still lacking, no clear indication exists on which imaging technique (MRI or CT scan) should be preferred, and no significant prognostic or predictive factors have been identified to distinguish between patients with indolent tumors and those who would need active therapy [3,31].

The rarity of the disease, the ubiquity of anatomical localizations, and the complexity of approach, which includes regular comparative radiological evaluations and critical interpretations of clinical behavior, are all factors that necessarily require a multidisciplinary approach in a referral center with specific expertise.

## 7. Description of Our Case Series

We retrospectively analyzed the data of 70 adult patients (34 females/36 males) with a proven diagnosis of *APC*-related FAP, referred to our center (Istituti Fisioterapici Ospedalieri (IFO) Regina Elena, Lazio, Italy) from January 2010 to March 2019.

All of the patients underwent a prophylactic colectomy and were subsequently followed through periodic follow-up examinations, including contrast-enhanced abdominal CT and MRI, depending on tumor location and status. The timing of surveillance was defined as MRI every 6–12 months in patients who developed DTs or tissue thickening; MRI every 2–3 months in patients with IAD and local infiltration, and both MRI and CT after 24–36 months in patients without DTs.

A total of 13/70 patients (18.6%), five females and eight males, were diagnosed with DTs, six DTs were already present during the first radiological examination and seven developed de novo during the follow-up period. Mean age at diagnosis was 31.2 years (range: 19–53) and mean time of onset after colectomy was 19.8 months (range: 9–34). In terms of location, two patients developed an AWD (15.4%), nine developed an IAD (69.2%), and two patients had lesions on both sites (15.3%). Overall, an IAD location was present in 11 cases (84.6%) (Table 2).

Based on the predisposing factors reported in previous studies, we observed that 10/13 (76.9%) patients had a positive familiar history for DTs; all patients underwent total colectomy with rectum preservation and ileorectal anastomosis; 7/13 patients received an open (53.8%) and 6 (46.2%) a laparoscopic surgical intervention; 8/13 cases (61.5%) had *APC* gene germline mutation 3′ to codon 1399, although the most frequently detected was the 1068delTCAA (4/13 cases, 30.8%) (Table 3).

Focusing on the 11 patients with IAD, CT/MRI detected a small bowel compression with loop dilations in five cases (45.4%) and no radiological sign of compression/infiltration, nor symptoms in 6 (54.5%). Among the five with radiological signs of compression/infiltration, one male aged 31 years, and one female, aged 21 years, also had asymptomatic ureteral compressions, which were treated with stent placements. Both patients had a rapid and progressive growth of IAD DTs and developed recurrent abdominal pain and episodic sub-occlusion and, thus, started chemotherapy. The female subject required three lines of chemotherapy (doxorubicin and dacarbazine, second-line vinorelbine, and rechallenge with vinorelbine), whereas the male subject required a surgical intervention after developing enteroenteric fistula, following two lines of chemotherapy (methotrexate and vinorelbine and second-line doxorubicin). Another patient, among the five, with radiological evidence of small bowel compression had a very rapid growth of the DTs, causing a fast development of intestinal perforation, and needed an emergency surgery. The patient achieved a good partial response (>50% reduction) and stable disease after first-line chemotherapy with weekly methotrexate and vinorelbine for about 6 months, and parenteral nutrition support for several months. The remaining two out of the five patients with radiological signs of intestinal compression had a single episode of sub-occlusion treated conservatively with rapid improvement and did not develop any complication. Therefore, 3/11 (27.3%) IAD had an aggressive behavior. The AWD showed a stable disease and did not cause any symptom.

Based on these data, we conducted a preliminary assessment on possible predictive markers of tumor aggressiveness, by focusing on/evaluating imaging, size of DT, and growth pattern, and by measuring the “average monthly growth rate” (AMGR), defined as the rate between the difference of tumor size measured by RECIST criteria 1.1 in two subsequent examinations, divided by the time (in months) between one investigation and the following (Table 4). The highest AMGR values were detected in the two patients who required surgery: 0.75 in the patient with enteroenteric fistula and 1.83 in the one with intestinal perforation, but not in the female patient who underwent chemotherapy (0.25). In one out of the eight patients with asymptomatic–paucisymptomatic IAD, the DT size was 11.6 cm and the AMGR was 0.42. In all of the other IAD and AWD cases, AMGR was <0.1.

In our series, the prevalence of DTs was 18.6%, this is in line with previous studies [72]; whereas the occurrence of intra-abdominal location (4.6%), was slightly higher compared with literature data, probably because we enrolled asymptomatic FAP for a post-surgical surveillance program. The analysis of predisposing factors revealed that the majority of patients had a positive family history of DTs (76.9%) and an *APC* gene germline mutation 3′ to codon 1399 (61.5%), in line with literature data on risk factors for DTs [17]. With regards to surgery, we did not observe any difference related to the approach (open vs. laparoscopic) of surgical intervention, and we could not perform any analysis on type of intervention as all of the patients underwent total colectomy with rectum preservation.

The main objective of our study was to assess the utility and the timing of surveillance for DTs in asymptomatic FAP patients from surgery onwards. Overall, the surveillance protocol used in this series of patients allowed to detect early asymptomatic intestinal obstruction and ureteral compression in two out of three cases with “aggressive” IAD, improving clinical management. In the third case, the radiological evidence of infiltrating DTs and the development of perforation occurred in a very short time, thus, surgical intervention was the only treatment option. In our series, there was a high frequency of aggressive DTs—nearly double that reported in other studies. However, these data should be interpreted cautiously because of the small number of patients in our series.

The remaining eight patients without aggressive IAD and the two with AWD were scheduled for close surveillance. One of them had a DT, with a worrisome size of 11.6 cm, which is a risk factor for developing complications [18], as reported by Quintini et al., and two had radiological signs of compression, none developed clinical complications. In summary, the radiological examination scheduled allowed a prompt intervention in the 3/11 aggressive IAD DT cases (27.2%) and an active surveillance of the remaining cases, avoiding unnecessary treatment despite the worrisome features detected in three cases.

Taking into account the unpredictable course of this benign, but locally aggressive tumor, recent position papers by the European Desmoid Working Group and of the European Organization for Research and Treatment of Cancer (EORTC)/Soft Tissue and Bone Sarcoma Group (STBSG)/Sarcoma Patients EuroNet (SPAEN) consider active surveillance with a regular instrumental monitoring (MRI or CT scan, depending on the anatomical site of origin of DT) from 1 to 3 months from baseline, and every 3–6 months thereafter, the “first-line” approach to asymptomatic DTs, independently from the sites of origin and from dimensions. The decision to start a treatment should rely only upon the documentation of a confirmed progression of disease on more than one instrumental reevaluation, with the only exceptions being worsening of symptoms or specific anatomic sites, as mesentery/abdominal cavity or head–neck district. In these latter cases, an active treatment (local or systemic) should be considered at the first documentation of progression of disease [31]. Considering the already described clinical behavior of the disease and the high risk of relapse after surgery, surgical approach should be considered the first option only in limited clinical situations, such as pictures of intestinal occlusion or perforation, for cosmetic concerns, in order to preserve a patient’s quality of life, or in case of localization, of the abdominal wall, due to the low morbidity and reported low risk of relapse.

In patients with FAP, the matter is the utility to start (or not) a surveillance after colectomy in asymptomatic and, likely negative, patients. The recently published UK guidelines suggest counseling patients about the risk of postoperative desmoid formation on the basis of genotype and family history, and to perform, in these cases, a radiological screening at 12 months following surgery [73]. The American College of Gastroenterology guidelines do not consider any radiological surveillance, but “evaluation is done for palpable masses and a full work-up for suggestive symptoms and a preoperative abdominal CT scan before colectomy may be considered if desmoids have been an issue in family members” [74]. The Mallorca Group [75] and the National Cancer Comprehensive Network [76] (NCCN) guidelines do not give specific recommendations.

On this basis, we believe that the AMGR index developed by our group could be useful as a tool to identify, in an asymptomatic phase, IAD’s that can develop aggressive behavior, becoming life-threatening and requiring active treatment.

From the preliminary results of our study, we can speculate that a high (>0.5) AMGR should be indicative of a very aggressive/life-threatening behavior, and requires a quick active treatment. A very low value (<0.1) is likely to be associated to stable DTs and follow-up could be delayed. AMGR values from 0.1 to 0.5 are a “grey area” that should be carefully observed with a close follow-up and monitoring of clinical symptoms. These preliminary results suggest that average tumor growth could be relevant in the evaluation of disease aggressiveness, whereas mesenteric thickening does not seem to be a sign of aggressive behavior. Certainly, further studies are needed to confirm this hypothesis and to identify a cut-off or range risk.

## 8. Conclusions

Active surveillance protocols are an important tool in the management of patients with DTs, although clear guidelines and possibly predictive markers to choose the most appropriate therapeutic path for each patient are still needed.

In our experience on patients with FAP-related DTs, an active surveillance strategy, with scheduled follow-up evaluations through abdominal CT scan/MRI, depending on tumor location and tissue thickening, was a valuable means to distinguish patients who needed prompt intervention from patients who presented apparently worrisome features, but did not require immediate treatment. We also identified a possible predictive marker of tumor aggressiveness called AMGR, which could distinguish patients with very aggressive/life-threatening tumor behavior (AMGR >0.5) from those with stable DTs (AMGR < 0.1).

Further studies are needed to confirm the relevance of AMGR and to develop other markers and clear guidelines to guide DTs treatment. Moreover, it is important to consider that, given the rarity of the disease, the ubiquity of anatomical localizations, and the complexity of approach that includes regular comparative radiological evaluations and critical interpretations of clinical behavior, are all factors that necessarily require a multidisciplinary approach in a referral center with specific expertise.

## Figures and Tables

**Table 1:** Characteristics of DTs in FAP vs. non FAP (sporadic) cases.

Characteristics	FAP-Related DT	Non-FAP (Sporadic) DT
Female (range)	45.4–54%	63.1–73.2%
Age at diagnosis (range); years	30.5–36	41.6–42.3
Location	51–68% IAD	50–89.1% EAD + AWD
Risk of recurrence	44%	25%
Mortality	14%	–

AWD: abdominal wall DT; DT: desmoid tumor; EAD: extra-abdominal DT; FAP: familial adenomatous polyposis; IAD: intra-abdominal DT.

**Table 2:** Characteristics of patients enrolled.

Characteristics	*n*	%
Total number of patients	70	100
Patients status:		
Presence of DT	13	18.6
Tissue thickening	18	25.7
Negative	39	55.7
Desmoid familiar history (yes/no)	16/54	23/77
Gender (male/female)	36/34	51/49
Age at colectomy (years), median (min–max)	24 (14–67)	
Type of surgery:		
Open	46	66
Laparoscopy	24	34

**Table 3:** Characteristics of patients who developed a desmoid tumor.

Characteristics	*n*	%
Total number of patients	13	
Age at diagnosis (years)	31.2 (19–53)	
Time between surgery and desmoid onset (available only for 10/13 patients) (months), median (min–max)	19.8 (9–34)	
Desmoid familiar history (yes/no)	10/3	76.9/23.1
Gender (male/female)	8/5	62/38
Age at colectomy (years), median (min–max)	26 (17–52)	
Type of surgery:		
Open	7	53.8
Laparoscopy	6	46.2
DT localization:		
AWD	2	15.4
IAD	9	69.2
Both	2	15.4

**Table 4:** Growth index among patients with desmoid tumor.

Patient	DT Site	Age (years)	Genotype	Size T0 cm	Size T1 cm	AMGR	Radiological Signs	Recurrent Abdominal Pain/Sub-Occlusion
1	IAD	34	c.2801delC	8.5	14	1.83	Loop dilation	Yes
2	AWD	41	c.3471-3474delGAGA	2	2	0	–	–
3	IADAWD	23	1068delTCAA	8	9	0.25	Loop dilation Hydronephrosis	Yes
				4	4			
4	IAD	nd	Exon duplication from 1 to 5	2.6	2.6	0	Loop dilation	No
				2	2			
5	IAD	21	c.3471-3474delGAGA	6	6	0	No	No
6	IAD	nd	1865delA	3.5	4.5	0.07	Loop dilation	No
7	IAD	nd	1865delA	3	3	0	no	No
				2	2			
8	IAD	30	1068delTCAA	7.3	7.3	0	no	No
9	IAD	29	1068delTCAA	3	3	0	no	No
10	AWD	19	1068delTCAA	2	2	0	–	–
11	IAD	53	c.2446_2456del11	6.5	11.6	0.42	no	No
12	IAD	nd	c2633delT	2	2.	0.02	no	No
13	IADAWD	31	c2633delT	14	17	0.75	Loop dilation Hydronephrosis	Yes
				3	3	0		

AMG: average monthly growth rate; AWD: abdominal wall desmoid; DT: desmoid tumor; IAD: intra-abdominal desmoid; nd: not determined; T0: time at diagnosis; T1: time at first follow-up examination.

## References

[1] De Marchis M., Tonelli F., Quaresmini D., Lovero D., Della-Morte D., Silvestris F., Guadagni F., Palmirotta R. (2017). Desmoid Tumors in Familial Adenomatous Polyposis. Anticancer. Res..

[2] Kasper B., Ströbel P., Hohenberger P. (2011). Desmoid Tumors: Clinical Features and Treatment Options for Advanced Disease. Oncoligs.

[3] Bonvalot S., Desai A., Coppola S., Le Péchoux C., Terrier P., Dômont J., Le Cesne A. (2012). The treatment of desmoid tumors: A stepwise clinical approach. Ann. Oncol..

[4] Houdt W.J., Husson O., Patel A., Jones R.L., Smith M.J.F., Miah A.B., Messiou C., Moskovic E., Al-Muderis O., Benson C. (2019). Outcome of Primary Desmoid Tumors at All Anatomic Locations Initially Managed with Active Surveillance. Ann. Surg. Oncol..

[5] Wong S.L. (2008). Diagnosis and management of desmoid tumors and fibrosarcoma. J. Surg. Oncol..

[6] Lips D.J., Barker N., Clevers H., Hennipman A. (2009). The role of APC and beta-catenin in the aetiology of aggressive fibromatosis (desmoid tumors). Eur. J. Surg. Oncol..

[7] Penel N., Coindre J.-M., Bonvalot S., Italiano A., Neuville A., Le Cesne A., Terrier P., Ray-Coquard I., Ranchère-Vince D., Robin Y.-M. (2016). Management of desmoid tumours: A nationwide survey of labelled reference centre networks in France. Eur. J. Cancer.

[8] Kasper B. (2020). The EORTC QLQ-C30 Summary Score as a Prognostic Factor for Survival of Patients with Cancer: A Commentary. Oncologist.

[9] Bertario L., Russo A., Sala P., Eboli M., Giarola M., D’Amico F., Gismondi V., Varesco L., Pierotti M., Radice P. (2001). Genotype and phenotype factors as determinants of desmoid tumors in patients with familial adenomatous polyposis. Int. J. Cancer.

[10] Campos F.G., Martinez C.A.R., Novaes M., Nahas S.C., Cecconello I., Figueiredo F.G.C.M.N. (2015). Desmoid tumors: Clinical features and outcome of an unpredictable and challenging manifestation of familial adenomatous polyposis. Fam. Cancer.

[11] Church J.M. (2008). Adenoma Detection Rate and the Quality of Colonoscopy: The Sword has Two Edges. Dis. Colon Rectum.

[12] Groen E.J., Roos A., Muntinghe F.L., Enting R.H., De Vries J., Kleibeuker J.H., Witjes M.J.H., Links T.P., Van Beek A.P. (2008). Extra-Intestinal Manifestations of Familial Adenomatous Polyposis. Ann. Surg. Oncol..

[13] Koskenvuo L., Ristimäki A., Lepistö A. (2017). Comparison of sporadic and FAP-associated desmoid-type fibromatoses. J. Surg. Oncol..

[14] Nieuwenhuis M.H., Casparie M., Mathus-Vliegen L.M.H., Dekkers O.M., Hogendoorn P.C.W., Vasen H.F. (2011). A nation-wide study comparing sporadic and familial adenomatous polyposis-related desmoid-type fibromatoses. Int. J. Cancer.

[15] Fallen T., Wilson M., Morlan B., Lindor N.M. (2006). Desmoid Tumors–A Characterization of Patients Seen at Mayo Clinic 1976–1999. Fam. Cancer.

[16] Schiessling S., Kihm M., Ganschow P., Kadmon G., Büchler M.W., Kadmon M. (2013). Desmoid tumour biology in patients with familial adenomatous polyposis coli. BJS.

[17] Sinha A., Tekkis P., Gibbons D.C., Phillips R.K., Clark S.K. (2010). Risk factors predicting desmoid occurrence in patients with familial adenomatous polyposis: A meta-analysis. Color. Dis..

[18] Quintini C., Ward G., Shatnawei A., Xhaja X., Hashimoto K., Steiger E., Hammel J., Uso T.D., Burke C.A., Church J.M. (2012). Mortality of Intra-Abdominal Desmoid Tumors in Patients With Familial Adenomatous Polyposis. Ann. Surg..

[19] Escobar C., Munker R., Thomas J.O., Li B.D., Burton G.V. (2011). Update on desmoid tumors. Ann. Oncol..

[20] Nieuwenhuis M.H., Mathus-Vliegen E.M., Baeten C.G., Nagengast F.M., Van Der Bijl J., Van Dalsen A.D., Kleibeuker J.H., Dekker E., Langers A.M., Vecht J. (2011). Evaluation of management of desmoid tumours associated with familial adenomatous polyposis in Dutch patients. Br. J. Cancer.

[21] Saito Y., Hinoi T., Ueno H., Kobayashi H., Konishi T., Ishida F., Yamaguchi T., Inoue Y., Kanemitsu Y., Tomita N. (2016). Risk Factors for the Development of Desmoid Tumor After Colectomy in Patients with Familial Adenomatous Polyposis: Multicenter Retrospective Cohort Study in Japan. Ann. Surg. Oncol..

[22] Nieuwenhuis M.H., Lefevre J.H., Bülow S., Järvinen H., Bertario L., Kernéis S., Parc Y., Vasen H.F.A. (2011). Family History, Surgery, and APC Mutation Are Risk Factors for Desmoid Tumors in Familial Adenomatous Polyposis: An International Cohort Study. Dis. Colon Rectum.

[23] Vitellaro M., Sala P., Signoroni S., Radice P., Fortuzzi S., Civelli E.M., Ballardini G., Kleiman D.A., Morrissey K.P., Bertario L. (2014). Risk of desmoid tumours after open and laparoscopic colectomy in patients with familial adenomatous polyposis. BJS.

[24] Hartley J.E., Church J.M., McGannon E., Fazio V.W. (2004). Significance of incidental desmoids found during surgery for familial adenomatous polyposis. Dis. Colon Rectum.

[25] Lazar A.J.F., Tuvin D., Hajibashi S., Habeeb S., Bolshakov S., Mayordomo-Aranda E., Warneke K.L., Terrada D.L., Pllock R.E., Lev D. (2008). Specific mutations in the beta-catenin gene (CTNNB1) correlate with local recurrence in sporadic desmoid tumors. Am. J. Pathol..

[26] Jasperson K.W., Patel S.G., Ahnen D.J., Adam M.P., Ardinger H.H., Pagon R.A., Wallace S.E., Bean L.J.H., Stephens K., Amemya A. (1998). APC-associated polyposis conditions. GeneReviews [Internet].

[27] Wehrli B.M., Weiss S.W., Yandow S., Coffin C.M. (2001). Gardner-Associated Fibromas (GAF) in Young Patients. Am. J. Surg. Pathol..

[28] Kotiligam D., Lazar A.J.F., Pollock R.E., Lev D. (2008). Desmoid tumor: A disease opportune for molecular insights. Histol. Histopathol..

[29] MacDonald B.T., Tamai K., He X. (2009). WNT/beta-catenin signaling: Components, mechanisms, and diseases. Dev. Cell.

[30] Slowik V., Attard T.M., Dai H., Shah R., Septer S. (2015). Desmoid tumors complicating Familial Adenomatous Polyposis: A meta-analysis mutation spectrum of affected individuals. BMC Gastroenterol..

[31] Kasper B., Baumgarten C., Garcia J., Bonvalot S., Haas R., Haller F., Hohenberger P., Penel N., Messiou C., Van Der Graaf W. (2017). An update on the management of sporadic desmoid-type fibromatosis: A European Consensus Initiative between Sarcoma PAtients EuroNet (SPAEN) and European Organization for Research and Treatment of Cancer (EORTC)/Soft Tissue and Bone Sarcoma Group (STBSG). Ann. Oncol..

[32] Mullen J.T., Delaney T.F., Kobayashi W.K., Szymonifka J., Yeap B.Y., Chen Y.-L., Rosenberg A.E., Harmon D.C., Choy E., Yoon S.S. (2012). Desmoid Tumor: Analysis of Prognostic Factors and Outcomes in a Surgical Series. Ann. Surg. Oncol..

[33] Van Broekhoven D.L.M., Verhoef C., Elias S.G., Witkamp A.J., Van Gorp J.M.H.H., Van Geel B.A.N., Wijrdeman H.K., Van Dalen T. (2013). Local recurrence after surgery for primary extra-abdominal desmoid-type fibromatosis. BJS.

[34] Cates J.M., Stricker T.P. (2014). Surgical resection margins in desmoid-type fibromatosis: A critical reassessment. Am. J. Surg. Pathol..

[35] Salas S., Dufresne A., Bui B., Blay J.-Y., Terrier P., Ranchere-Vince D., Bonvalot S., Stoeckle E., Guillou L., Le Cesne A. (2011). Prognostic Factors Influencing Progression-Free Survival Determined from a Series of Sporadic Desmoid Tumors: A Wait-and-See Policy According to Tumor Presentation. J. Clin. Oncol..

[36] Bonvalot S., Eldweny H., Haddad V., Rimareix F., Missenard G., Oberlin O., Vanel D., Terrier P., Blay J., Le Cesne A. (2008). Extra-abdominal primary fibromatosis: Aggressive management could be avoided in a subgroup of patients. Eur. J. Surg. Oncol..

[37] Penel N., Le Cesne A., Bonvalot S., Giraud A., Bompas E., Rios M., Salas S., Isambert N., Boudou-Rouquette P., Honore C. (2017). Surgical versus non-surgical approach in primary desmoid-type fibromatosis patients: A nationwide prospective cohort from the French Sarcoma Group. Eur. J. Cancer.

[38] Van Broekhoven D.L., Deroose J.P., Bonvalot S., Gronchi A., Grünhagen D.J., Eggermont A.M.M., Verhoef C. (2014). Isolated limb perfusion using tumour necrosis factor a and melphalan in patients with advanced aggressive fibromatosis. Br. J. Surg..

[39] Kujak J.L., Liu P.T., Johnson G.B., Callstrom M.R. (2009). Early experience with percutaneous cryoablation of extra-abdominal desmoid tumors. Skelet. Radiol..

[40] AIOM Giudelines. https://www.aiom.it/wp-content/uploads/2019/10/2019_LG_AIOM_Sarcomi-1.pdf.

[41] Cates J.M. (2015). Prognostic factors for second recurrence after surgical resection of recurrent desmoid-type fibromatosis. Pathol. Oncol. Res..

[42] Merchant N.B., Lewis J.J., Woodruff J.M., Leung D.H., Brennan M.F. (1999). Extremity and trunk desmoid tumors: A multifactorial analysis of outcome. Cancer.

[43] Latchford A.R., Sturt N.J.H., Neale K., Rogers P.A., Phillips R.K.S. (2006). A 10-year review of surgery for desmoid disease associated with familial adenomatous polyposis. BJS.

[44] Gega M., Yanagi H., Yoshikawa R., Noda M., Ikeuchi H., Tsukamoto K., Oshima T., Fujiwara Y., Gondo N., Tamura K. (2006). Successful Chemotherapeutic Modality of Doxorubicin Plus Dacarbazine for the Treatment of Desmoid Tumors in Association with Familial Adenomatous Polyposis. J. Clin. Oncol..

[45] Seow-Choen F. (2008). The management of desmoids in patients with familial adenomatous polyposis (FAP). Acta Chir. Iugosl..

[46] Fiore M., Rimareix F., Mariani L., Domont J., Collini P., Le Péchoux C., Casali P.G., Le Cesne A., Gronchi A., Bonvalot S. (2009). Desmoid-Type Fibromatosis: A Front-Line Conservative Approach to Select Patients for Surgical Treatment. Ann. Surg. Oncol..

[47] Desurmont T., Lefevre J.H., Shields C., Colas C., Tiret E., Parc Y. (2014). Desmoid tumour in familial adenomatous polyposis patients: Responses to treatments. Fam. Cancer.

[48] Ray M.E., Lawrence T.S., Redston M., Bertagnolli M.M. (2006). Radiation Therapy for Aggressive Fibromatosis (desmoid tumor). J. Clin. Oncol..

[49] Keus R.B., Nout R.A., Blay J.-Y., De Jong J.M., Hennig I., Saran F., Hartmann J.T., Sunyach M.P., Gwyther S.J., Ouali M. (2013). Results of a phase II pilot study of moderate dose radiotherapy for inoperable desmoid-type fibromatosis—An EORTC STBSG and ROG study (EORTC 62991–22998). Ann. Oncol..

[50] Janssen M.L., Van Broekhoven D.L.M., Cates J.M., Bramer W.M., Nuyttens J.J., Gronchi A., Salas S., Bonvalot S., Grünhagen D.J., Verhoef C. (2017). Meta-analysis of the influence of surgical margin and adjuvant radiotherapy on local recurrence after resection of sporadic desmoid-type fibromatosis. BJS.

[51] Lev-Chelouche D., Abu-Abeid S., Nakache R., Issakov J., Kollander Y., Merimsky O., Meller I., Klausner J.M., Gutman M. (1999). Limb desmoid tumors: A possible role for isolated limb perfusion with tumor necrosis factor-alpha and melphalan. Surgery.

[52] Picariello L., Brandi M.L., Formigli L., Orlandini S.Z., Dolara P., Caderni G., Raimondi L., Tonelli F. (1998). Apoptosis induced by sulindac sulfide in epithelial and mesenchymal cells from human abdominal neoplasms. Eur. J. Pharmacol..

[53] Wang Y.-C., Wong J.-U. (2016). Complete remission of pancreatic head desmoid tumor treated by COX-2 inhibitor—A case report. World J. Surg. Oncol..

[54] De Camargo V.P., Keohan M.L., D’Adamo D.R., Antonescu C.R., Brennan M.F., Singer S., Ahn L.S., Maki R.G. (2010). Clinical outcomes of systemic therapy for patients with deep fibromatosis (desmoid tumor). Cancer.

[55] Hansmann A., Adolph C., Vogel T., Unger A., Moeslein G. (2004). High-dose tamoxifen and sulindac as first-line treatment for desmoid tumors. Cancer.

[56] Nishida Y., Tsukushi S., Shido Y., Wasa J., Ishiguro N., Yamada Y. (2010). Successful Treatment with Meloxicam, a Cyclooxygenase-2 Inhibitor, of Patients With Extra-Abdominal Desmoid Tumors: A Pilot Study. J. Clin. Oncol..

[57] Picariello L., Tonelli F., Brandi M.L. (2004). Selective oestrogen receptor modulators in desmoid tumours. Expert Opin. Investig. Drugs.

[58] Libertini M., Mitra I., Van Der Graaf W.T.A., Miah A.B., Judson I., Jones R.L., Thomas K., Moskovic E., Szucs Z., Benson C. (2018). Aggressive fibromatosis response to tamoxifen: Lack of correlation between MRI and symptomatic response. Clin. Sarcoma Res..

[59] Palassini E., Frezza A.M., Mariani L., Lalli L., Colombo C., Fiore M., Messina A., Casale A., Morosi C., Collini P. (2017). Long-term Efficacy of Methotrexate Plus Vinblastine/Vinorelbine in a Large Series of Patients Affected by Desmoid-Type Fibromatosis. Cancer J..

[60] Skapek S.X., Ferguson W.S., Granowetter L., Devidas M., Perez-Atayde A.R., Dehner L.P., Hoffer F.A., Speights R., Gebhardt M.C., Dahl G.V. (2007). Vinblastine and Methotrexate for Desmoid Fibromatosis in Children: Results of a Pediatric Oncology Group Phase II Trial. J. Clin. Oncol..

[61] Constantinidou A., Jones R.L., Scurr M., Al-Muderis O., Judson I. (2009). Pegylated liposomal doxorubicin, an effective, well-tolerated treatment for refractory aggressive fibromatosis. Eur. J. Cancer.

[62] Garbay D., Le Cesne A., Penel N., Chevreau C., Marec-Berard P., Blay J.-Y., Debled M., Isambert N., Thyss A., Bompas E. (2011). Chemotherapy in patients with desmoid tumors: A study from the French Sarcoma Group (FSG). Ann. Oncol..

[63] Chugh R., Wathen J.K., Patel S., Maki R.G., Meyers P.A., Schuetze S.M., Priebat D.A., Thomas D., Jacobson J.A., Samuels B.L. (2010). Efficacy of Imatinib in Aggressive Fibromatosis: Results of a Phase II Multicenter Sarcoma Alliance for Research through Collaboration (SARC) Trial. Clin. Cancer Res..

[64] Dufresne A., Bertucci F., Penel N., Le Cesne A., Bui B., Tubiana-Hulin M., Ray-Coquard I., Cupissol D., Chevreau C., Perol D. (2010). Identification of biological factors predictive of response to imatinib mesylate in aggressive fibromatosis. Br. J. Cancer.

[65] Gounder M.M., Lefkowitz R.A., Keohan M.L., D’Adamo D.R., Hameed M., Antonescu C.R., Singer S., Stout K., Ahn L., Maki R.G. (2011). Activity of Sorafenib against Desmoid Tumor/Deep Fibromatosis. Clin. Cancer Res..

[66] Gounder M.M., Mahoney M.R., Van Tine B.A., Ravi V., Attia S., Deshpande H.A., Gupta A.A., Milhem M., Conry R.M., Movva S. (2018). Sorafenib for Advanced and Refractory Desmoid Tumors. N. Engl. J. Med..

[67] Toulmonde M., Ray-Coquard I.L., Pulido M., Andre T., Isambert N., Chevreau C., Penel N., Bompas E., Thyss A., Bertucci F. DESMOPAZ Pazopanib (PZ) Versus IV Methotrexate/Vinblastine (MV) in Adult Patients with Progressive Desmoid Tumors (DT) a Randomized Phase II Study from the French Sarcoma Group (Abstract). https://meetinglibrary.asco.org/record/160662/abstract.

[68] Alman B., Attia S., Baumgarten C., Benson C., Blay J.-Y., Bonvalot S., Breuing J., Cardona K., Casali P.G., Van Coevorden F. (2020). The management of desmoid tumours: A joint global consensus-based guideline approach for adult and paediatric patients. Eur. J. Cancer.

[69] Healy J.C., Reznek R.H., Clark S.K., Phillips R.K., Armstrong P. (1997). MR appearances of desmoid tumors in familial adenomatous polyposis. Am. J. Roentgenol..

[70] Tonelli F., Picariello L., Nesi G., Franchi A., Valanzano R., Asteria C.R., Brandi M.L. (2006). Desmoid tumours in familial adenomatous polyposis. Inflammatory Bowel Disease and Familial Adenomatous Polyposis.

[71] Crago A.M., Denton B., Salas S., Dufresne A., Mezhir J.J., Hameed M., Gonen M., Singer S., Brennan M.F. (2013). A Prognostic Nomogram for Prediction of Recurrence in Desmoid Fibromatosis. Ann. Surg..

[72] Pikaar A., Nortier J.W.R., Griffioen G., Vasen H.F.A. (2002). Desmoid tumors in patients with familial adenomatous polyposis. Ned. Tijdschr Geneeskd..

[73] Monahan K.J., Bradshaw N., Dolwani S., DeSouza B., Dunlop M.G., East J.E., Ilyas M., Kaur A., Lalloo F., Latchford A. (2019). Guidelines for the management of hereditary colorectal cancer from the British Society of Gastroenterology (BSG)/Association of Coloproctology of Great Britain and Ireland (ACPGBI)/United Kingdom Cancer Genetics Group (UKCGG). Gut.

[74] Syngal S., Brand R.E., Church J.M., Giardiello F.M., Hampel H.L., Burt R.W. (2015). ACG Clinical Guideline: Genetic Testing and Management of Hereditary Gastrointestinal Cancer Syndromes. Am. J. Gastroenterol..

[75] Vasen H.F., Moslein G., Alonso A., Aretz S., Bernstein I., Bertario L., Blanco I., Bülow S., Burn S.J., Capellá G. (2008). Guidelines for the clinical management of familial adenomatous polyposis (FAP). Gut.

[76] NCCN Guidelines. www.nccn.org.

